# Antimicrobial Usages and Antimicrobial Resistance in Commensal *Escherichia coli* From Veal Calves in France: Evolution During the Fattening Process

**DOI:** 10.3389/fmicb.2019.00792

**Published:** 2019-04-12

**Authors:** Emilie Gay, Maxime Bour, Géraldine Cazeau, Nathalie Jarrige, Christophe Martineau, Jean-Yves Madec, Marisa Haenni

**Affiliations:** ^1^Université de Lyon – ANSES, Laboratoire de Lyon, Unité Épidémiologie et Appui à la Surveillance, Lyon, France; ^2^Université de Lyon – ANSES, Laboratoire de Lyon, Unité Antibiorésistance et Virulence Bactériennes, Lyon, France; ^3^Centre National de Référence de la Résistance aux Antibiotiques, Centre Hospitalier Universitaire de Besançon, Besançon, France; ^4^IDELE, Service Qualité des Viandes, Le Rheu, France

**Keywords:** *E. coli*, resistance, ESBL, CTX-M, veal calves

## Abstract

Extended-Spectrum-Cephalosporin (ESC)-resistant Enterobacteriaceae have widely spread in all settings worldwide. In animals, Extended-Spectrum Beta-Lactamase (ESBL) producers have been frequently identified in veal calves. The objectives of this study were to investigate the trends in the ESBL load and antimicrobial resistance (AMR) proportions, and antimicrobial usages (AMU) in veal calves during the fattening process. Ten fattening farms were selected and 50 animals per farm were sampled. AMR was assessed in bacteria from the dominant flora (collected on non-selective MacConckey agar) and in ESBL/AmpC-carrying bacteria from the subdominant flora (selected on ChromID ESBL selective plates) upon arrival and 5–6 months later before slaughter. The number and types of treatments during fattening were also collected. Rates of ESBL-producing *E. coli* from the subdominant flora significantly decreased in all farms (arrival: 67.7%; departure: 20.4%) whereas rates of multidrug-resistant *E. coli* from the dominant flora have significantly increased (arrival: 60.2%; departure: 67.2%; *p* = 0.025). CTX-M-1 was the most frequently identified ESBL enzyme (arrival: 59.3%; departure: 52.0%). The plasmid-mediated *mcr-1* gene was also identified occasionally. In parallel, levels of resistances to non-critically important antimicrobials were already high upon arrival but have still further increased over time until slaughter. Our study also highlighted that if only ESBL-producing isolates were monitored, it might have led to a partial (and partly false) picture of AMR rates globally decreasing during the fattening period. The mean number of antimicrobial treatments per calf (NTPC) was 8.75 but no association between AMU and AMR was evidenced. Most ESBL producers were clonally unrelated suggesting multiple sources and not cross-contaminations among calves during transportation. Feeding milk containing antimicrobial residues to veal calves is hypothesized to explain the high ESBL loads in animals at the entrance on farms.

## Introduction

Extended-Spectrum-Cephalosporin (ESC)-resistant Enterobacteriaceae have widely spread in the human, animal and environmental reservoirs thanks to the epidemic success of Extended-Spectrum and AmpC Beta-Lactamase (ESBLs/AmpC) genes, plasmids and clones. Even though the distribution of ESC resistance genes in different settings still differs to a certain extent, such as the *bla*_CTX-M-15_ ESBL gene mostly identified in humans, horizontal gene transfers across sectors have also been documented extensively, making the whole epidemiological picture more and more complex. In this respect, the food chain has been regarded as a potential source of human colonization/infection by ESC-resistant Enterobacteriaceae (Leverstein-van Hall et al., 2011; Kluytmans et al., 2013; Huijbers et al., 2014). In particular, chicken has often been incriminated because of the high proportions of ESC-resistant *E. coli* isolates detected in broilers, including in chicken meat at retail worldwide (Efsa Panel on Biological Hazards et al., 2012; Borjesson et al., 2013; Dierikx et al., 2013; Casella et al., 2017). Nonetheless, a recent study in the Netherlands showed that other meat types (principally beef, but also pork and veal) may play an important role in contaminating humans with ESBL producers through under-cooked meat and/or cross-contamination in the kitchen (Evers et al., 2017). In all, for risk assessment purposes, data are required from all sectors along the food chain, i.e., from the birth of the animals in farms up to the different food processing and consumption steps.

Veal calves grown for meat production are frequently exposed to antibiotics (Pardon et al., 2012; Jarrige et al., 2017). Carriage of ESBL/AmpC producers is also frequent in veal calves compared to adults (Madec et al., 2008; Schmid et al., 2013), as shown by recent prevalence data of around 30 and 40% of ESBL-producing *E. coli* colonizing veal calves at slaughterhouse in France and the Netherlands, respectively (Madec et al., 2008; Hordijk et al., 2013c). Such an ESBL/AmpC load in veal calves is surely worrying, and whereas reducing the global antimicrobial use (AMU) in the veal calves sector is undoubtedly of utmost importance, in-depth investigations are also needed to clarify the origins and causes of such elevated proportions of resistances. A study by Hordijk et al. on the within-farm dynamics showed that the proportion of calves carrying an ESBL-producing *E. coli* was around 20% upon arrival on the fattening farm, and then decreased to close to zero over a 10 weeks period (Hordijk et al., 2013a). A limitation of the study is that only three farms were included, but these results were in accordance with a recent EFSA scientific opinion on the risk of antimicrobial resistance (AMR) associated with feeding milk containing antimicrobial residues to calves.

The aim of the present longitudinal study was first to expand the existing knowledge on ESBL/AmpC producers from the subdominant flora carried by veal calves by monitoring the proportion of ESC-resistant *E. coli* in these animals upon arrival at the fattening farm and just before their departure to the slaughterhouse. In parallel, the second goal was to assess AMR phenotypes in bacteria from the dominant flora at the same time points. Ten unrelated farms were selected and 50 animals per farms were monitored for ESBL/AmpC carriage and other AMR phenotypes. We also collected the number and types of treatments received during fattening process in order to correlate AMU with AMR, and identify possible intervention strategies to reduce the AMR burden in the veal calves sector.

## Materials and Methods

### Study Design and Data Collection

Ten veal calves farms belonging to the Idele network were included in the study that started in November 2013. The inclusion criteria were to have a minimum herd size of 50 calves and to raise a single batch (group of calves entering the farm at the same time and reared together until slaughter). The inclusion was based on a voluntary basis. A total of 50 animals were selected and sampled twice per batch (rectal swab), once upon arrival at the farm and just before departure to slaughterhouse, approximately 5 months later. A questionnaire allowed to collect information on farm characteristics (geographical location, other food animals reared on the farm), housing conditions (herd size, number of calves per pen, housing ventilation system, floor type), feeding system, all-in all-out process (duration, cleaning and disinfection) and description of the batch (number of calves, breed, dates of arrival and departure, average calf weight upon arrival, use of oral rehydration solution upon arrival, mortality). Veterinary prescriptions and dates of treatments recorded in the farm health register were gathered for each batch to estimate AMU.

### Bacteria Isolation and Identification

Rectal swabs were sent to the Anses Lyon laboratory within 24 h after sampling and processed upon arrival. They were directly plated in parallel: (i) onto MacConkey agar (bioMérieux, Marcy l’Etoile, France) for the culture of the dominant flora and (ii) onto selective ChromID ESBL agar (bioMérieux) for the selection of ESC-resistant isolates from the subdominant flora. After incubation at 37°C for 24 h, one presumptive *E. coli* colony was arbitrary selected from each plate and isolates were identified using mass spectrometry through Matrix Assisted Laser Desorption Ionization Time-Of-Flight (MALDI-TOF). Should the isolate not be identified as an *E. coli*, another colony was selected and identified. The process was repeated up to three times if needed.

### Antimicrobial Susceptibility Testing

Antimicrobial susceptibility was tested using the disk diffusion method on Mueller-Hinton agar and results were interpreted according to the breakpoints recommended by the Antibiogram committee of the French society of microbiology^1^. The antibiotics tested were eight beta-lactams (amoxicillin, amoxicillin-clavulanic acid, cefalotin, cefuroxime, ceftiofur, cefoxitin, cefquinome, and ertapenem) and eight non-beta-lactams (tetracycline, gentamicin, streptomycin, florfenicol, colistin, sulfonamides, nalidixic acid, and enrofloxacin). The *E. coli* ATCC 25922 strain was used as quality control. Florfenicol was tested for epidemiological purposes using the breakpoints assigned for *Pasteurella* spp. For all isolates presenting an intermediate diameter for colistin, the minimum inhibitory concentration (MIC) was determined using the microdilution method. ESBL production was determined by the double-disc synergy test as recommended by EUCAST.

### Identification of β-Lactamase-Encoding and Plasmid-Mediated Genes

The DNA of each isolate was extracted by boiling a colony for 10 min in H_2_0 (500 μl). PCRs were performed using specific primers for the detection of *bla*_CTX-M_ group 1, group 9, group 2, and *bla*_CMY_ genes (Shibata et al., 2006; Dierikx et al., 2010). For all *bla*_CTX-M_ group 1, additional PCRs were performed using the primers ISEcp1L1/P2D. All positive amplicons were sequenced (Genewiz, London, United Kingdom). The detection of the *mcr-1* to *mcr-5* genes was performed using the recently published multiplex PCR (Rebelo et al., 2018).

### Phylogeny and Genetic Relatedness of ESBL-Producing Isolates

Phylogenetic grouping of the ESBL-producing *E. coli* isolates was performed using the improved method described by Doumith et al. (2012). Pulsed-field gel electrophoresis (PFGE) was performed on a subset of 10 ESBL-producing isolates per farm upon arrival and all ESBL-producing *E. coli* isolates at departure using the restriction enzyme *XbaI*. DNA fingerprints were analyzed using the Dice correlation coefficient, with tolerance and optimization set at 0.5 and 1%, respectively (BioNumerics, Ghent, Belgium).

### Statistical Analysis

Proportion of resistance for each antibiotic was calculated as the number of animals harboring a resistant *E. coli* divided by the total number of animals tested. Comparisons of proportions were done using the Chi-squared test. The significance level was set to 0.05. Within-farm proportions were also calculated.

Multi-resistance was defined as the resistance to at least three antibiotics from different antimicrobial groups, and the seven molecules used to assess this multi-resistance were amoxicillin, ceftiofur, gentamicin, florfenicol, tetracycline, sulfonamides and enrofloxacin.

AMU was assessed by estimation of the number of antimicrobial treatments per calf (NTPC) over the fattening period using the same methodology as Jarrige et al. (2017). Total NTPC and class-specific NTPC were calculated.

The associations between AMR of commensal *E. coli* isolates and farm management factors – including AMU – were assessed through linear models, one for each combination of *E. coli* (dominant flora or ESC-resistant flora) – antibiotic tested (16 antibiotics). The statistical unit was the farm and the dependant variable was the difference between within-farm proportion of resistance at departure and upon arrival. The putative risk factors used as explanatory variables were all the variables collected in the questionnaire plus AMU, this latter variable being tested successively through the total NTPC and the class-specific NTPC.

### Ethics Statements

This study was declared to the CNIL, the French office responsible for protecting personal data, supporting innovation and preserving individual liberties. No further ethical approval was needed since this study did not involve any experimentation on animals (only rectal swabs were sampled) and since we did not collect and register any personnel opinion of the participants.

## Results

### Studied Sample

A total of 498 animals from 10 farms (designated farm A to J) were sampled upon arrival (50 calves sampled per farm, except for farm C where only 48 animals have been sampled due to local constraints). This first sampling period spanned from November 2013 to February 2014. All animals that were still alive at the time of slaughter (*n* = 481/498, 96.6%) were sampled during the week before slaughter; this second sampling period spanned from April to July 2014.

The mean batch size was 338 calves, the mean weight of calves upon arrival was 53 kg and the fattening period lasted 160 days on average. The breed of veal calves were cross-breed (from dairy cows and beef bulls) for 40% of the batches (4/10), Holstein for 30%, a mix of Holstein and mixed breed (Normand or Montbéliard) for 20% and a mix of Holstein and cross-breed for 10%. All the farms housed calves on slatted floors. The feeding system was buckets or trough in 8 of the 10 farms (80%), in which the calves were grouped in pens of 2–5 animals. In farm H, the feeding system was an automatic milk distribution system and the calves were grouped by 50. Farm C used a mix between buckets and automatic milk distribution system and the calves were grouped in pens of 18 animals.

### Evolution of AMR in *E. coli* Recovered From the Dominant Flora

An *E. coli* isolate was recovered from the non-selective MacConkey agar from all samples, meaning that *E. coli* were present in the dominant flora of all calves. Of the 979 isolates, 21 (2.1%) displayed an ESBL phenotype, 17 (3.4%) from the first sampling upon arrival (7 positive farms; between 1 and 5 positive animals by positive farm), 4 (0.8%) from the second sampling before departure (2 positive farms; one with 3 positive animals, the second with 1 positive animal). The ESBL phenotype was due to the presence of CTX-M group 1 (*n* = 10, all CTX-M-1), CTX-M group 9 (*n* = 9), and CTX-M group 2 (*n* = 2) enzymes. ESBL-producing *E. coli* belonged to the phylogroups A (*n* = 8), B1 (*n* = 5), and D (*n* = 8), while B2 was not identified. Proportions of resistances to amoxicillin, tetracyclines, streptomycin and sulfonamides were very high (>60%) at arrival of animals in the farm, and had significantly increased at departure (Table 1). Proportions of resistances to other beta-lactams than amoxicillin were overall low and significantly decreased during the fattening process. Resistance to quinolones also significantly decreased from arrival to departure. A total of 11 isolates were resistant to colistin (MICs ranging between 6 and 16 mg/L) of which 9 were detected in animals upon arrival (originating from 7 different farms), and 2 in animals at departure (both originating from the same farm). The *mcr-1* gene was detected in 4 of the 9 colistin-resistant isolates upon arrival, as well as in the two isolates at departure.

**Table 1:** Resistance of *E. coli* isolates from the dominant flora for the 16 antibiotics tested, upon arrival and before departure to slaughterhouse.

Antibiotic	Breakpoints (mm: S ≥ /R < )	Resistant isolates upon arrival (*n* = 498)		Resistant isolates at departure (*n* = 481)		p comparison arrival/departure
		Number	Proportion (%)	Number	Proportion (%)	
Amoxicillin	21/14	315	63.3	334	69.4	0.041
Amoxicillin – clavulanic acid	21/14	25	5.0	3	0.6	<0.001
Cefalotin	18/12	29	5.8	8	1.7	0.001
Cefuroxime	22/22	40	8.0	10	2.1	<0.001
Ceftiofur	21/18	19	3.8	4	0.8	0.002
Cefoxitin	22/15	3	0.6	1	0.2	0.332
Cefquinome	22/19	19	3.8	4	0.8	0.002
Ertapenem	28/26	0	0	0	0	–
Tetracycline	19/17	357	71.7	437	90.9	<0.001
Gentamicin	18/16	11	2.2	69	14.4	<0.001
Streptomycin	15/13	353	70.9	383	79.6	0.002
Florfenicol	19/15	38	7.6	26	5.4	0.153
Colistin	18/15	6	1.2	1	0.2	0.064
Sulfonamides	17/12	351	70.5	377	78.4	0.005
Nalidixic acid	20/15	115	23.1	49	10.2	<0.001
Enrofloxacin	19/19	46	9.2	26	5.4	0.022


The proportion of multi-resistant isolates significantly increased from 60.2% upon arrival to 67.2% at departure of animals (*p* = 0.025). The proportion of isolates susceptible to the seven selected antibiotics was 23.3% upon arrival and 7.3% at departure (Supplementary Table S1). Only two isolates displayed co-resistances to all seven antibiotics.

### Evolution of ESC-Resistances and Co-resistances in *E. coli* Recovered From the Subdominant Flora

**FIGURE 1:**
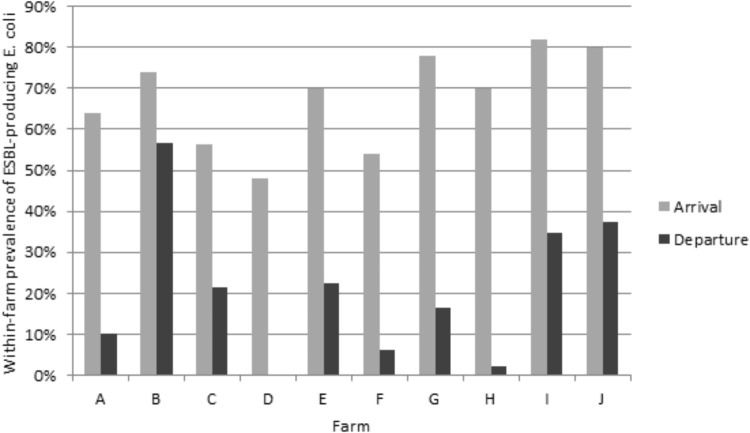
Within-farm proportion of ESBL-producing *E. coli* (selective medium) upon arrival and at departure of animals.

Of the 979 samples, 437 (44.7%) ESC-resistant *E. coli* were isolated from the subdominant flora on the selective plates. The ESBL phenotype was confirmed for 435 (44.4%) *E. coli* isolates, as shown by individual antimicrobial susceptibility testing and double-disc synergy test. The two remaining *E. coli* isolates presented an AmpC phenotype, which was confirmed by the detection of the *bla*_CMY -2_ gene. The proportion of ESBL-producing *E. coli* significantly decreased from 67.7% (337/498) upon arrival of animals to 20.4% (98/481) at departure (*p* < 0.001). The within-farm proportion of ESBL-producing *E. coli* ranged from 48.0 to 82.0% upon arrival of the veal calves in the fattening farm, and from zero to 56.5% at departure to the slaughterhouse (Figure 1). The ESBL phenotype was largely due to the presence of CTX-M group 1 enzymes, which were identified in 71.5% of the animals upon arrival, and in 61.2% upon departure to the slaughterhouse (Table 2). Of the 241 *bla*_CTX-M-group1_–carrying *E. coli* upon arrival, 200 harbored *bla*_CTX-M-1_ (200/241, 83.0%), 23 *bla*_CTX-M-15_ (23/241, 9.5%), 12 *bla*_CTX-M-32_ (12/241, 5.0%)_,_ 4 *bla*_CTX-M-55_ (4/241, 1.7%), and 2 *bla*_CTX-M-3_ (2/241, 0.8%). The PFGE profiles performed on a subset of 10 ESBL-producing isolates per farm upon arrival showed a wide variability without any clustering (data not shown). At departure, *bla*_CTX-M-1_ was also the most frequently identified gene (51/60, 85%) followed by *bla*_CTX-M-55_ (4/60, 6.7%), *bla*_CTX-M-15_ (3/60, 5.0%), and *bla*_CTX-M-3_ (2/60, 3.3 %). At departure, the PFGE profiles were much more similar than upon arrival so that a high degree of clonality was observed inside each farm (Supplementary Table S2). As an example, Supplementary Figure S1 shows the PFGE distribution in farm E at departure, where three distinct PFGE profiles were observed. It highlights the epidemiological success of certain ESBL *E. coli* clones more than others during the fattening process. Nonetheless, since different CTX-M enzymes were also produced by the same clone (Supplementary Figure S1, lanes 1–4), not only a clonal but also a plasmid dissemination has likely occurred, which illustrates the complexity of ESBL spread at farm level. Similarly, the emergence of CTX-M-2 enzymes before slaughter was most likely due to the dissemination of a single clone within farm C since all but one CTX-M-2 enzymes were identified in this farm. Altogether, depending on the farm presenting ESBL-positive isolates, from 1 (farm H, 1 ESBL-producing *E. coli* isolate) to 7 (farm B, 26 ESBL-producing *E. coli* isolates) distinct PFGE profiles were observed at the end of the fattening process (Supplementary Table S2). Of note, none of the successful clones identified at departure for the slaughterhouse was shared between farms, proving a specific and local evolution. *E. coli* belonged to phylogroups A (*n* = 134, 39.8%), B1 (*n* = 78, 23.1%), B2 (*n* = 9, 2.7%), and D (*n* = 116, 34.4%) upon arrival, and to phylogroups A (*n* = 50, 51.0%), B1 (*n* = 15, 15.3%), B2 (*n* = 1, 1.0%), and D (*n* = 32, 32.7%) at departure to slaughterhouse.

**Table 2:** Characterization of ESBL-producing isolates.

Farm	ESBL-producing isolates upon arrival (*n* = 337)				ESBL-producing isolates at departure (*n* = 98)			
	Number	CTX-M-			Number	CTX-M-		
		Group 1	Group 9	Group 2		Group 1	Group 9	Group 2	
A	32	26	6	0	5	3	2	0
B	37	30	7	0	26	5	0	21
C	27	17	10	0	10	2	8	0
D	24	14	10	0	0	0	0	0
E	35	28	7	0	11	8	3	0
F	27	20	7	0	3	1	1	1
G	39	31	8	0	8	8	0	0
H	35	34	1	0	1	1	0	0
I	41	31	10	0	16	16	0	0
J	40	10	26	4	18	16	2	0
Total	337	241	92	4	98	60	16	22
Percentage	100.0	71.5	27.3	1.2	100.0	61.2	16.3	22.5


Proportions of co-resistances to tetracyclines, streptomycin and sulfonamides were very high (>80%) at arrival, but resistances to streptomycin had significantly decreased at departure (74.5%) whereas the two others stayed approximately at the same level (Table 3). Proportions of co-resistances to enrofloxacin were also significantly lower at departure compared to arrival. Upon arrival, 25 colistin-resistant isolates (MICs ranging between 6 and 24 mg/L) were detected, which originated from 8 different farms. The *mcr-1* gene was identified in 18 isolates, while one isolate carried both the *mcr-1* and *mcr-3* genes. At departure for slaughterhouse, only 4 animals from 2 different farms still carried a colistin-resistant *E. coli* (MICs ranging between 2 and 4 mg/L). The *mcr-3* gene was detected in all four isolates and was co-harbored with the *bla*_CTX-M-55_ gene.

**Table 3:** Resistance phenotype associated to the 435 ESBL-producing *E. coli* isolates from the subdominant flora (selective medium), upon arrival and before departure to slaughterhouse.

Antibiotic	Resistant isolates upon arrival (*n* = 337)		Resistant isolates at departure (*n* = 98)		p comparison arrival/departure
	Number	Proportion (%)	Number	Proportion (%)
Tetracycline	299	88.7	91	92.9	0.212
Gentamicin	33	9.8	13	13.3	0.325
Streptomycin	287	85.2	73	74.5	0.014
Florfenicol	35	10.4	5	5.1	0.111
Colistin	12	3.6	0	0	0.123
Sulfonamides	324	96.1	94	95.9	1.000
Nalidixic acid	134	39.8	30	30.6	0.099
Enrofloxacin	78	23.2	9	9.2	0.002


### Antimicrobial Use

**FIGURE 2:**
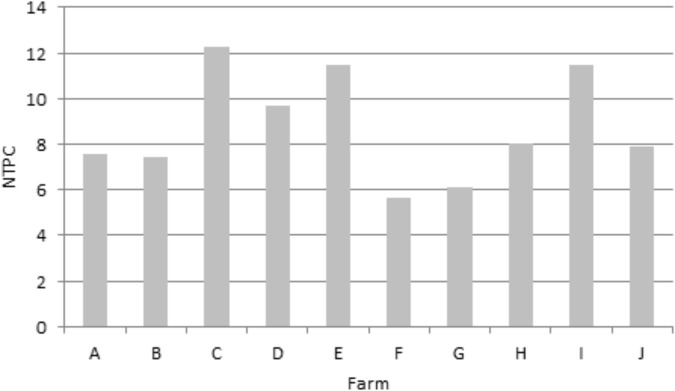
Mean number of treatments per calf (NTPC) over the fattening period for each farm.

The mean NTPC was 8.75 meaning that, on average, a calf received 8.75 antimicrobial treatments during the fattening period. The minimum NTPC was 5.65 and the maximum 12.24 (Figure 2). The majority (98%) of these treatments were group treatments (administered to the whole batch). The antimicrobial class most prescribed was tetracyclines (NTPC = 4.97), followed by polypeptides (1.64), sulfonamides (0.79) and macrolides (0.65). The third and fourth generation cephalosporins accounted for 0.04 treatment per calf. AMU by antimicrobial class is detailed for each farm in Supplementary Table S3. No antimicrobial other than antibiotics (such as heavy metals) were used in the farms.

### Risk Factors for Antimicrobial Resistance

No significant association between AMR in commensal *E. coli* isolates and farm management factors (including AMU) was evidenced, whatever the *E. coli* population, the AMR phenotype or the indicator of AMU considered.

## Discussion

This study firstly showed that the proportion of ESBL-producing *E. coli* carriage in the sub-dominant gut flora of veal calves was considerably high upon arrival on fattening farms, ranging from 48.0 to 82.0%. Such a very high fecal shedding of ESBL producers most likely reflects their selection in the farms where calves were born. Another explanation refers to possible cross-transfers of ESBL-positive *E. coli* clones or ESBL plasmids among animals during transportation but, as shown by PFGE, the diversity of ESBL-producing *E. coli* isolates in veal calves at arrival rather suggests multiple sources. Other hypotheses include the use of antibiotics for the treatment of gastroenteritis in newborns, which is a frequent disease during the days after birth, or cross-contamination from the farm environment (Randall et al., 2014). Notably however, these data are also in accordance with a recent scientific opinion from EFSA highlighting the risk for the development of AMR due to feeding calves with waste milk, i.e., of treated cows that were milked during the withdrawal period, which is a common practice in dairy farms in Europe.

Our study also shows the complex interplay between clonal and plasmid transmission of resistant traits, such as ESBL genes. Each farm evolved independently, with a strong reduction in the number of PFGE patterns and the success of a limited number of ESBL *E. coli* clones which were over-represented at the end of the fattening period. Interestingly, none of the farms shared the same successful clones. Moreover, as shown in Supplementary Figure S1, plasmid spread can also be suggested when identical clones harbored two different ESBL genes.

### Decrease of the Proportion of ESBL-Producing Bacteria From the Subdominant Flora During the Fattening Process

In all farms, the rate of ESBL carriage as detected on selective plates has strongly decreased during the fattening process, from 67.71% upon arrival to 20.41% at departure. We observed the same trend reported in the Netherlands by Hordijk et al. (2013a), but with a different sampling design since we studied 10 different farms (versus three farms in the Netherlands) over a period of around 23 weeks (versus 10 weeks). Interestingly, such a decrease of ESBL-producers has also been reported during the pig production cycle (Hansen et al., 2013) whereas, on the contrary, an increase in ESBL/AmpC-producers has been reported in broiler fattening farms (Laube et al., 2013), a divergence that may reflect specificities of each food-producing sector. In the present study, the within-farm proportion of ESBL-producing *E. coli* remained above 10% for the majority of farms (6 out of 10) and only one had a proportion equal to zero. This is also in contrast with the Dutch study where 2 out of the 3 farms reached zero and the last one 1.4%. This difference may partly be due to the large confidence interval around the within-farm proportion considering that both studies enrolled a limited number of farms.

The decreasing dynamic of ESBL-producing *E. coli* carriage can be partly explained by the age of the animals, as several studies showed that in young dairy calves, animal age was negatively associated with proportion of AMR in the gut flora (Hoyle et al., 2004a,b). The reduction in the number of PFGE patterns and the success of a limited number of clones may also partly explain this decreasing trend. Nevertheless, even though the proportion of ESBL producers has decreased, it still remained higher than the one reported in adult cattle. This decreasing dynamic could also be partly due to the rare occurrence of treatments with ESCs during the fattening process. Indeed, ESCs were used only in 6/10 farms and accounted for the lower NTPC. On the contrary, first-line antibiotics such as tetracyclines, which largely accounted for the higher NTPC, can select or co-select ESBL producers due to the frequent co-localization of those genes on the same genetic platforms, and thus possibly explain that ESBL-producing *E. coli* never completely disappeared in the farms studied. However, tetracyclines obviously did not contribute to a broad expansion of ESBL-producing isolates, which could be attributed to major differences in bacterial population sizes within the digestive tract of calves between the large non-ESBL dominant flora and the more limited ESBL subdominant flora. Since both *E. coli* populations were widely resistant to tetracyclines, a major positive selective impact of the use of tetracyclines on the specific spread of ESBL producers would likely have little chance to occur.

### Increase of the Proportion of Non-critically Important Antibiotics in Bacteria From the Dominant Flora During the Fattening Process

An interesting observation is the high and increasing levels of resistances to non-critically important antimicrobials in the dominant flora, contrary to the low and decreasing proportion of ESBL-producing isolates. Such a trend has also occurred in parallel to a significant increase in proportion of multi-resistant *E. coli* isolates between the arrival of veal calves and their departure for slaughterhouse. It proves that the average number of 8.8 treatments administered to each calf throughout the fattening process can still select for resistant isolates, even though proportions of resistances were already high upon arrival. The important NTPC demonstrates a high level of AMU but critically important antimicrobials were only rarely prescribed. In this study, which was performed between 2013 and 2014, colistin was frequently used. The current situation is substantially different since colistin use has drastically decreased in 2016 in France after the discovery of the plasmid-borne transmissible *mcr* gene and the governmental measures taken to limit the spread of this resistance determinant. The evaluation of the impact on AMR of such a major change in antibiotic prescription albeit focused on the single drug class of polymyxins would need novel investigations.

### Predominance of the CTX-M-1 Enzyme

Molecular data obtained in this study confirmed *bla*_CTX-M-1_ as the main gene responsible for the spread of ESBLs in veal calves. This is also consistent with the global ESBL picture observed in food-producing animals in France, where *bla*_CTX-M-1_ has been identified in poultry, cattle and pigs (Meunier et al., 2006; Girlich et al., 2007; Dahmen et al., 2012; Casella et al., 2017; Baron et al., 2018; Lucas et al., 2018). A high prevalence of *bla*_CTX-M-1_ has also been reported in the same veal calves sector in other European countries, such as the Netherlands (Hordijk et al., 2013a,b,c). More globally, *bla*_CTX-M-1_ is a dominant ESBL gene in several animal species in Europe and the data presented here most probably reflect the most common selection pathway of ESBL producing *E. coli* in the animal sector in this continent. Nonetheless, the emergence of ESBL genes that are atypical in the bovine sector in Europe, such as *bla*_CTX-M-55_ or *bla*_CTX-M-2_, will have to be monitored, in line with the epidemic success of *bla*_CTX-M-2_ in this study and with the recent description of *bla*_CTX-M-55_-producing *E. coli* co-producing the *rmtB* or *mcr-3* genes in veal calves (Haenni et al., 2018; Lupo et al., 2018). Phylogroup detection showed that *E. coli* isolates from healthy veal calves largely belonged to commensals from group A or B1. The potentially pathogenic group D of *E. coli* has also been identified in around one third of the isolates whereas the B2 group was rare, and B2 isolates never belonged to the human epidemic ST131 strain.

### Absence of Association Between Antimicrobial Use and Resistance

Several other studies demonstrated no association between AMU and AMR (Agga et al., 2016; Gonggrijp et al., 2016; Kylie et al., 2017; Santman-Berends et al., 2017). On the contrary, other studies in different animal productions evidenced this association (Pereira et al., 2014; Makita et al., 2016; Andersen et al., 2017; Scott et al., 2018). Particularly, Catry et al. (2016) highlighted a strong relation between antimicrobial treatment incidences and resistance profiles of *E. coli* strains in veal calves. But the two studies did not use the same indicators, especially for AMR. Catry et al. (2016) used an AMR index combining resistance to all antibiotics for a single strain, whereas we used separate indicators for each antibiotic. But the main difference is that we decided to use the farm as the statistical unit and the difference between within-farm proportion of resistance at departure and upon arrival, and not only one single measure of resistance. This allowed focusing on the phenomenon during the fattening process and especially AMU during this period. The side effect is a lack of power since only 10 farms were monitored, and since the difference of within-farm resistance between arrival and departure of calves could be low (already high levels upon arrival of animals). But we can also have another hypothesis to explain this lack of association: AMU was very high globally, all the animals were exposed to AMU, and this may have limited the ability to identify any effect of AMU as AMR selection has even occurred in the less exposed farms (Benedict et al., 2015).

However, despite this limitation, our results provide important data on AMU in the veal calves sector and the evolution dynamic of ESBL-producing *E. coli* isolates, multi-drug resistant *E. coli* isolates as well as *E. coli* isolates resistant to non-critically important antibiotics over the fattening period.

## Conclusion

Our study has shown that ESBL-producing *E. coli* from the subdominant flora were on a decreasing trend in veal calves in 10 unrelated fattening farms, pointing out that those animals had most likely been contaminated beforehand during their first days of life. While pressure to decrease antibiotic use have to be maintained in fattening farms, our study showed that even more efforts have to be put in the farms of origin of the veal calves, in order to further decrease the number of ESBL-positive individuals entering the fattening process. In parallel, levels of resistances to non-critically important antimicrobials in bacteria from the dominant flora were already high upon arrival but have still further increased over time until slaughter. Consequently, our study also clearly highlighted that if only ESBL-producing isolates from the subdominant flora were monitored, it might have led to a partial (and partly false) picture of AMR rates globally decreasing during the fattening period. These results should promote surveillance systems not only relying on a single AMR indicator (such as the prevalence of ESBL-producers) but, where possible, to implement a more global approach of AMR monitoring.

## Author Contributions

EG, MH, and J-YM designed the experiments and analyzed the data. CM supervised the sampling campaign. MB, GC, EG, and NJ performed the experiments. MH and EG drafted the manuscript. J-YM actively contributed to the manuscript’s writing. All authors approved the final version of this manuscript.

## Conflict of Interest Statement

The authors declare that the research was conducted in the absence of any commercial or financial relationships that could be construed as a potential conflict of interest.

## Footnotes

1 https://www.sfm-microbiologie.org/
