# Supplementary material for: Antimicrobial Usages and Antimicrobial Resistance in Commensal Escherichia coli From Veal Calves in France: Evolution During the Fattening Process
Source: Front Microbiol. 2019 Apr 12;10:792. doi: 10.3389/fmicb.2019.00792 (PMC6473463; doi:10.3389/fmicb.2019.00792)
Supplement: Supplementary file 1 [file Data_Sheet_1.PDF]

Table S1. Co-resistance of *E. coli* isolates from the dominant flora among the 7 antibiotics considered for multi-resistance analysis, upon arrival and before departure to slaughterhouse.

Number of antibiotics presenting a resistant phenotype[1]	Resistant isolate upon arrival (n=498)		Resistant isolate at departure (n=481)	
	Number	Proportion (%)	Number	Proportion (%)
0	116	23.3	35	7.3
1	26	5.2	43	8.9
2	56	11.3	80	16.6
3	230	46.2	257	53.4
4	46	9.2	32	6.7
5	21	4.2	33	6.9
6	1	0.2	1	0.2
7	2	0.4	0	0.0

[1]The seven antibiotics considered are amoxicillin, ceftiofur, gentamicin, florfenicol, tetracycline, sulfonamides and enrofloxacin.

Table S2. Number of PFGE patterns at departure to slaughterhouse.

Farm	No. of ESBL-producing isolates at departure	No. of different PFGE patterns
A	5	2
B	26	7
C	10	4
D	0	0
E	11	4
F	3	3
G	8	2
H	1	1
I	16	4
J	18	5

Table S3. Mean number of treatments per calf (NTPC) in each farm over the fattening period, by antimicrobial class.

Farm	Total NTPC per farm	NTPC for [a] :								
		AG	C3/C4G	FQ	Macrolides	Penicillins	Phenicols	Polypeptides	Sulfonamides	TET
A	7.56	0	0.08	0.04	0.15	0	0.09	1.80	0.78	4.62
B	7.40	0.23	0.05	0	0.03	0.11	0.07	1.04	0.54	5.32
C	12.24	0.01	0.11	0	0.13	0.40	0.07	2.36	0.90	8.27
D	9.66	0.73	0	0.17	0.68	0.12	0.06	1.24	1.24	5.42
E	11.48	0.86	0.10	0.05	1.23	0.27	0.06	1.23	1.01	6.66
F	5.65	0.46	0	0.06	0.07	0.04	0.02	1.25	0.53	3.22
G	6.12	0.12	0	0	0.55	0.48	0.02	2.21	0	2.73
H	8.05	0.01	0.01	0	1.56	0.04	0.08	1.51	0.89	3.95
I	11.46	0.49	0	0.03	1.94	0.19	0.06	0.94	1.01	6.81
J	7.91	0.03	0.07	0.12	0.18	0.95	0.08	2.81	1.01	2.67
Mean NTPC per antibiotic class	8.75	0.29	0.04	0.05	0.65	0.26	0.06	1.64	0.79	4.97

[a]Antibiotic class: AG, aminoglycosides; C3/C4G, 3rd and 4th generation cephalosporins; FQ, fluoroquinolones; TET, tetracyclines.

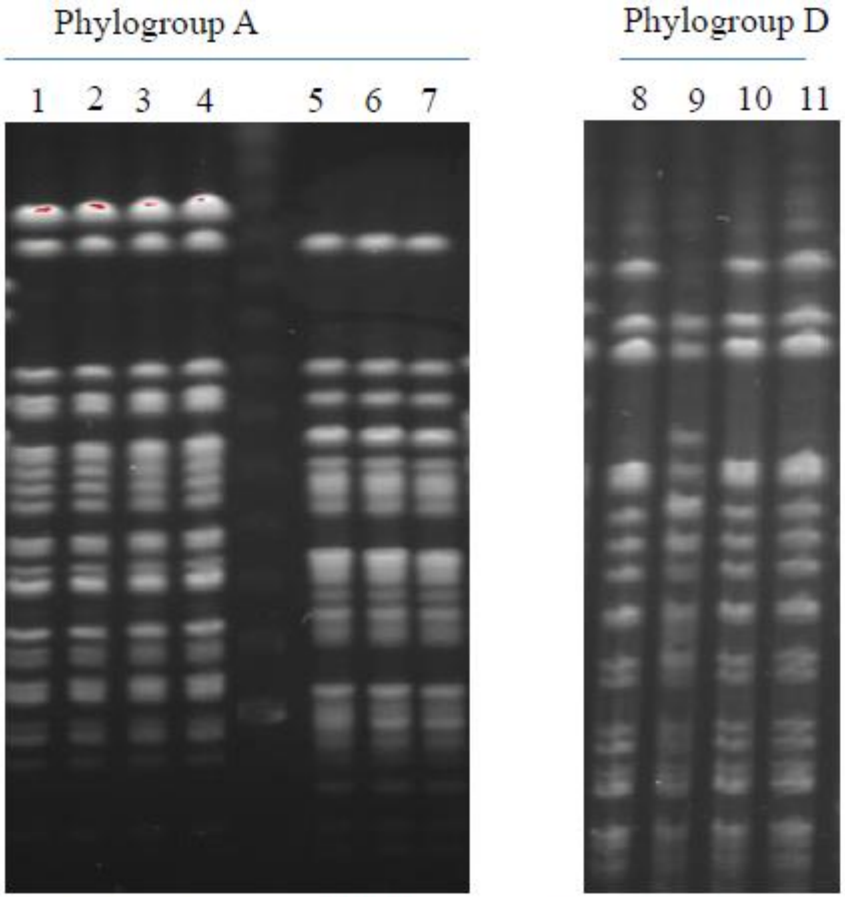

Figure S1. PFGE patterns of the 11 ESBL isolates collected in farm E at departure for slaughterhouse. 1, CTX-M-15; 2-4, CTX-M-1; 5-7, CTX-M-9; 8-10, CTX-M-1.

The clonal dissemination can clearly be observed since patterns from a same farm are identical or similar. Plasmid transmission can also be suggested in the case of isolate 1 (CTX-M-15) and isolates 2-4 (CTX-M-1), which share identical PFGE profiles but different CTX-M enzymes.
